# Soil-Transmitted Helminthic Infections and Geophagia among Pregnant Women in Jimma Town Health Institutions, Southwest Ethiopia

**DOI:** 10.4314/ejhs.v31i5.16

**Published:** 2021-09

**Authors:** Mestawet Getachew, Ruth Yeshigeta, Abebaw Tiruneh, Yonas Alemu, Eden Dereje, Zeleke Mekonnen

**Affiliations:** 1 School of Pharmacy, Institute of Health, Jimma University; 2 School of Medical Laboratory Sciences, Institute of Health, Jimma University; 3 Molecular Biology Research Center, Jimma University

**Keywords:** Pregnant women, Geophagia, STHs, Jimma, Ethiopia

## Abstract

**Background:**

Pregnancy is a key step for human's reproduction and continuity of generation. Pregnant women are among at risk groups for the infection of soil-transmitted helminths (STHs). STHs are highly prevalent in low- and middle-income countries due to the deprived environmental sanitation and personal hygiene. Eating soil (geophagia) is also commonly practiced by pregnant women, particularly in developing countries. The aim of this study was to determine the prevalence of STHs and geophagia, and to assess associated factors among pregnant women in Jimma, Southwest Ethiopia.

**Methods:**

A cross sectional study was conducted among 407 pregnant women attending antenatal care (ANC) at different health facilities located in Jimma Town. Data related to sociodemographic and geophagia practice was collected using a structured questionnaire and STH infections status was determined by using McMaster technique.

**Results:**

A total of 407 pregnant women were included in this study. The overall prevalence of any STHs was 19.7% (80/407). Ascaris lumbricoides was the most prevalent 45(56.2%), followed by Trichuris trichiura 19(23.8%) and hookworms 12(15%). There were 4(5%) of double infection with A. lumbricoides and T. trichiura. Overall, 71 (17.4%) of the pregnant women responded to practice geophagia. STHs infection was significantly higher among geophagic pregnant women (p<0.01) and pregnant women who practiced geophagia were 3 times more likely (OR 2.9, 95% CI 1.3–4.2) to have the STHs compared to non-geophagic. Out of those who claimed soil eating habits, 59.1% preferred reddish soil type. Geophagia practice was significantly higher during the third trimester as compared to first and second (p<0.05).

**Conclusion:**

Geophagia is a risky behavior and this study showed a significant association of geophagia practice with STH infections, although the causal relation could not be established.

## Introduction

Pregnancy is a key step for human's reproduction and continuity of generation. During pregnancy, there are three fetal development stages known as first, second and third trimesters. Throughout these gestational stages different physiological and developmental changes take place in a mother and her fetus, respectively (1,2). Food craving, food aversion, and pica (intentional consumption of non-food materials including soil) are common during pregnancy due to hormonal fluctuations and other non-specific reasons (3,4). Immunological changes during pregnancy may also increase the susceptibility of pregnant women to variety of infections including soil-transmitted helminths (STHs) (5). The STHs group comprises *A. lumbricoides, T. trichiura* and hookworms (*Ancylostoma duodenale* and *Necator americanus*). These four species of helminthic parasites need soil contact mandatorily for the development of infective stages or continuity of their life cycle (6). More than two billion people believed to be infected globally with STHs. The disease burden is higher in low- and middleincome countries due to the deprived environmental sanitation and personal hygiene. Particularly, school children and pregnant women are among at high risk groups. In Sub-Sahara Africa, an estimated of 7 million, 173 million and 162 million pregnant women already infected with hookworms, *A. lumbricoides* and *T. trichiura*, respectively. STH infections during pregnancy might result in maternal anemia and problematic birth outcomes. Particularly, hookworm infections result in iron deficiency anemia which is an important contributor of maternal morbidities and mortalities. The impact of infected mothers also imposes negative birth outcomes including low birth weight, still birth, and preterm delivery, particularly in endemic regions of the globe (7–11). Ethiopia also exhibited high prevalence of STHs among pregnant women and studies showed significant association with anemia during pregnancy (11–13).

On the other hand, geophagia (intentional earth/soil eating) is also commonly practiced by pregnant women in developing countries. Self-reported reasons for geophagia include enjoyment of soil taste, texture and smell, and it is also a kind of culture to show pregnancy in some African countries (14–16). Pregnant women claimed the benefit of soil eating as it gives relief from morning sickness related nausea and stomach upsets (17,18). Studies also showed clay fraction of soil (19) protects gastrointestinal tract disturbance and/or infections by directly adsorbing distress causing agents, reenforcing the luminal epithelium by absorbing liquids, and lysing bacterial cells (20–23). However, soil may also harbor toxic materials and infectious organisms. Consumption of contaminated soil during geophagia practice may therefore expose the pregnant women to toxic minerals, pathogenic microbes, and helminthic infections (24–28). Although the national level magnitude of geophagia is not yet known in Ethiopia, prevalence of pica and/or geophagia among pregnant women in some areas of the country like Sidama Zone and Addis Ababa was reported to be 30.4% and 20.3%, respectively (4,29).

Therefore, this study was aimed to determine the prevalence of STHs and geophagia practice, and to assess associated risk factors among pregnant women in Jimma Town, Southwest Ethiopia.

## Materials and Methods

**Study design and setting**: Health facility based cross sectional study was conducted from March to June, 2016 among pregnant women who were attending antenatal care (ANC) at three public health institutions namely; Jimma University Specialized Hospital, Jimma Health Center and Jimma Higher-2 Health Center, all located in Jimma Town, Southwest Ethiopia. Jimma town is located in Southwestern Ethiopia at 350 km from the capital city, Addis Ababa. The geographical locations of the town is 7°41′ N latitude and 36° 50′E longitude, 1780 m above sea level, and the annual rainfall and temperature are 1138 mm to 1690 mm and 30°C/14°C (maximum/minimum), respectively.

**Sample size and sampling**: The total sample size was calculated using single population proportion formula considering a previous prevalence of 41% STHs (13) and presuming a 5% non-response rate. Accordingly, a total of 407 consented pregnant women from the three health facilities during the study period were included as study participants. A convenient sampling method was employed where three groups of study team deployed to the three health facilities during the same period and enrolled the study participants until the calculated sample size reached.

**Data collection and analysis**: Sociodemographics, practice of geophagia and associated risk factors of geophagia and STH infections were assessed using semi-structured questionnaire which is administered verbally by interviewers (two pre-trained BSc nurses).

Study subjects were instructed to collect their own thumb sized stool sample and provided with tissue paper, applicator stick and clean, labeled and leak-proof stool cup. A wet mount was performed immediately for possible intestinal parasitic infections and the aliquots of stool samples were transported to Jimma University STHs laboratory using cold box. Single McMaster fecal egg counting technique (analytic sensitivity of 50 eggs per gram (EPG)) (30) was used for the detection and enumeration of *A. lumbricoides, T. trichiura* and hookworm eggs.

Questionnaire data were checked for their completeness each day, entered to Excel, and cleaned. Coded data was imported to STATA_MP version 12 (Stata Corp., TX, USA) for analysis. Using the software, descriptive results were tabulated and each independent variable was checked for possible significant association with geophagia and/or STHs using bivariate analysis. Odds ratio (OR) was used for statistical significance and p-value less than 0.05 was considered as statistically significant at 95% confidence interval.

**Ethics**: Ethical clearance was obtained from Ethical Review Board of Jimma University Institute of Health. Permission was obtained from Jimma Town Health Office and each health facility. The aim of the study was explained to participating pregnant women and written informed consent was obtained. Pregnant women infected with intestinal parasite(s) were treated in collaboration with the respective health facilities according to the national guidelines. Confidentiality of the data was maintained at all levels.

## Results

A total of 407 pregnant women were enrolled in this study: 120 from Jimma University Specialized Hospital, 140 from Jimma Health Center, and 147 from Jimma Higher-2 Health Center. The age of the pregnant women ranged from 15 to 39 years with a mean age of 24.7±4.8SD. From the total study participants, 227(55.8%) were primigravida (first pregnancy) and 180(44.2%) were multigravida (multiparous pregnancy or subsequent pregnancies). The number of pregnant women in the first, second and third trimesters were 90 (22.1%), 175 (43.0%) and 142 (34.9%), respectively (Table 1).

**Table 1:** Socio-demographics and geophagic habits of pregnant women in Jimma Town, Southwest Ethiopia

Characteristics		N (%)
Age	15–18	38(9.3)
	19–39	369(89.7)
Trimester	First	90 (22.1)
	Second	175 (43.0)
	Third	142 (34.9)
Residence	Rural	248(60.9)
	Urban	159(39.1)
Education	Literate	197(48.4)
	Illiterate	210(51.6)
Parity	Primigravida	227(55.8)
	Multigravida	180(44.2)
Consumption of	No	371(91.1)
other non-food	Yes	36(8.9)
materials		
Geophagic practice	Yes	71(17.4)
	No	336(82.6)

**Among pregnant women who experienced geophagia (n=71)**		

Reason for	Whimsical	28 (39.4)
geophagy	Smell	43 (60.6)
Soil texture	Clay (gray/black finely-grained natural rock or soil material)	14 (19.7)
	Loam (reddish soil)	42 (59.1)
	Chalky (whitish soil)	15 (21.2)
Frequency	Occasional	37 (52.1)
	Regular	34 (47.9)
Abdominal pain	No	50 (70.4)
after geophagy	Yes	21 (29.6)
(within 24 hrs.)		

From the total study participants, 80(19.7%) were positive for any STH infections. Among the STH species detected the most prevalent parasite was *A. lumbricoides*- 45(56.2%) followed by *T. trichiura*- 19(23.8%) and hookworms- 12(15%). Double infections with *A. lumbricoides* and *T. trichiura* were detected in 4 (5%) of pregnant women's stool sample. The intensity of all STHs species was light infections, 120–1360 EPG (egg per gram) (<4,999 EPG), 80–600 EPG (<999 EPG) and 40–360 EPG (<1,999 EPG) for *A. lumbricoides, T. trichiura* and hookworms, respectively. Using wet mount, only one *Giardia lamblia* and one *Schistosoma mansoni* cases were additionally detected. The sensitivity of wet mount was lower than McMaster technique and no extra cases of STHs were found by stool wet mount.

Infection with STHs did not show statistically significant association with socio-demographics and other related variables such as age groups, educational level and soil texture. There was statistically significant association between STH infections and geophagia with p-value < 0.01 and pregnant women who practiced geophagia were three times more likely (OR (95%CI): 2.9(1.3–4.2) to have the STHs compared to nongeophagic. However, frequency of soil eating and consumption of other non-food materials lack association with the STH infections (Table 2).

**Table 2:** Prevalence and risk factors of STHs among pregnant study participants in Jimma Town, Southwest Ethiopia

Characteristics		N (%)	STH		OR	95%CI	p-value

			No	Yes			
Age	15–18	38(9.7)	28(73.7)	10(26.3)	1.5	0.7–3.3	0.13
	19–39	369(90.7)	299(81)	70(19)	Ref		
Residence	Rural	248(60.9)	193(77.8)	55(22.2)	1.5	0.9–2.6	0.11
	Urban	159(39.1)	134(84.3)	25(15.7)	Ref		
Education	Literate	197(48.4)	158(80.2)	39(19.8)	Ref		
	Illiterate	210(51.6)	169(80.5)	41(19.5)	1.0	0.6–1.6	0.95
Gestation	1^st^	90(22.1)	71(78.9)	19(21.1)	1.2	0.6–2.3	0.6
	2^nd^	175(43)	140(80)	35(20)	1.1	0.6–2	0.7
	3^rd^	142(34.9)	116(81.7)	26(18.3)	Ref		
Geophagy	No	336(82.6)	279(83)	57(17)	Ref		
	Yes	71(17.4)	48(67.6)	23(32.4)	2.9	1.3–4.2	0.004*
Soil texture	Clay	14(19.7)	10(71.4)	4(28.6)	Ref		
	Loam	42(59.2)	31(73.8)	11(26.2)	0.9	0.2–3.4	0.86
	Chalky	15(21.1)	7(46.7)	8(53.3)	2.9	0.6–13.3	0.18
Geophagy	Occasional	37(52.1)	25(67.6)	12(32.4)	Ref		
Frequency	Regular	34(47.9)	23(67.6)	11(32.4)	1	0.4–2.7	0.99
Other non-	No	371(91.1)	299(80.6)	72(19.4)	Ref		
food materials	Yes	36(8.9)	28(77.8)	8(22.2)	1.2	0.5–2.7	0.68

* Significant association, OR: odds ratio

With respect to geophagia, out of the total study participants, 71(17.4%) had practice of intentional soil eating habit (geophagia), and from these, 37(52.1%) eat soil occasionally and 34(47.9%) regularly. Highly preferred soil type by the pregnant women was loam (reddish soil type just below the surface) followed by clay (gray and finely grained soil) and chalky (whitish soil type). The pregnant women guess that the collected soil for geophagy was clean and they did not process/bake the soil before eating. The reason for claiming geophagia by the pregnant women was due to soil smell and whimsical. The number of pregnant women who consumed other non-food materials including charcoal and coffee residue was 36(8.9%). Abdominal discomfort within the same day of eating soil was complained by 21 (29.6%) pregnant women (Table 1). On the other hand, geophagia had no statistically significant association with age groups, residence, marital status, educational level and parity. The trimester stage of the study participants had statistically significant association with the habit of geophagia in which pregnant women at their third trimester practice geophagia twice as compared to pregnant women at their first trimester, with p=0.04(OR 2.3, 95% CI 1.0–5.1) (Table 3).

**Table 3:** Associated risk factors of geophagia among pregnant study participants in Jimma Town, Southwest Ethiopia

Characteristics		N (%)	Geophagy		OR	95%CI	p-value

			No	Yes			
Age	15–18	38(59.71)	31(81.6)	7(18.4)	1.1	0.5–2.5	0.87
	19–39	369(40.29)	305(82.7)	64(17.3)	Ref		
Residence	Rural	248(60.93)	204(82.26)	44(17.74)	Ref		
	Urban	159(39.07)	132(83.02)	27(16.98)	0.9	0.6–1.6	0.8
Marital	Married	383(94.10)	319(83.29)	64(16.71)	Ref		
status	Unmarried	24(5.90)	17(70.83)	7(29.17)	2.1	0.8–5.2	0.13
Parity	1	227(55.77)	192(84.58)	35(15.42)	0.7	0.4–1.2	0.228
	>1	180(44.23)	144(80.00)	36(20.00)	Ref		
Education	Literate	197(48.40)	162(82.23)	35(17.77)	Ref		
	Illiterate	210(51.60)	174(82.86)	36(17.14)	1	0.6–1.6	0.9
Occupation	Housewife	253(62.16)	211(83.40)	42(16.60)	Ref		
	Others	154(37.84)	125(81.17)	29(18.83)	1.2	0.8–2.1	0.33
Trimester	First	90(22.11)	81(90.00)	9(10.00)	Ref		
	Second	175(43.00)	142(81.14)	33(18.86)	2.1	0.95–4.6	0.066
	Third	142(34.89)	113(79.58)	29(20.42)	2.3	1–5.1	0.04*

* Significant association, OR-odds ratio

## Discussion

The global burden of STHs is still significant with more than two billion people are already infected. Pregnant women are classified as at high risk group due to their susceptibility to the STH infections and devastating health outcomes (5,31). Infection with STHs during pregnancy imposes iron deficiency anemia, malnutrition, and sometimes serious complications and deaths of mothers. Their baby may also face problems such as still birth, low birth weight, and cognitive disorders after birth (7–10).

Ethiopia is among countries with high prevalence of STHs and pregnant women in the country suffer from health impacts of the infections (11). The finding of our study revealed that about 20% of pregnant study participants were infected with any STHs. Our result is much lower than the result of a study conducted around Gilgel Gibe dam which is located in rural area of Jimma zone which reported 41% prevalence of STHs among pregnant women (13). Studies conducted in northern and northwest Ethiopia also reported higher prevalence of STHs, 51.5% and 70.6%, respectively (32,33). Those variations might be attributed to differences in the socio-economic, environmental and personal hygiene of the study participants. Moreover, such prevalence variability in study result might be from epidemiological variations from place to place in Ethiopia (34). Another study conducted in western Ethiopia shows comparable prevalence (24.7%) of STHs with our findings(12). Deworming of pregnant women after the first trimester is recommended in regions where the prevalence of hookworms and/or *T. trichiura* is >20% and anemia is >40% among pregnant women (31). Despite high prevalence of STHs in different part of Ethiopia, there are no national implementation program (mass drug administration) targeting pregnant women. Therefore, deworming and health information for STHs during ANC follow-up are recommended in endemic areas of the country based on WHO recommendations.

On the other hand, food craving, food aversion and non-food material consumption (pica) are common habits of women during pregnancy (3). The smell of soil and whimsical were among various reasons given by the subjects for their geophagia practice. Study conducted in Southern Nations, Nationalities, and People's (SNNP) region in Ethiopia reported soil, soft stones, charcoal and coffee residue as highly preferred non-food materials by the pregnant women. Geophagia (soil eating) accounted for more than half of pica practice by the pregnant women participated in the study conducted in SNNP region (4). The result of our study showed 17.4% of pregnant women deliberately eat soil and 8.9% of pregnant women consume other non-food materials such as coffee residue and charcoal. The result of our study also showed significant association of geophagia with the trimester stages of the pregnancy, in which pregnant women in the third trimester had higher soil eating habits as compared to pregnant women in the first and second trimesters. This may be due to increased physiological demand of micronutrients for the growth of the fetus and/or hormonal effects during late gestational stages (29,35). On the other hand, our study was cross-sectional and assessed the prevalence of geophagia and not determined when they started soil eating. This might have influenced the number of geophagic pregnant women assessed during their third trimester.

Generally, the effect of geophagia remains debatable and it is reported as source of important minerals or toxics (17,27). It is also claimed as possible source of different infectious microorganisms such as helminthic infections, mainly *A. lumbricoides* and *T. trichiura,* because of their eggs remain viable for long period in the soil (28,36). Studies conducted for possible association between STHs and geophagia have controversial results. Study conducted in North Ethiopia reported significant association between geophagia and STHs (32), while other studies conducted elsewhere reported absence of significant association between geophagia and STHs (37,38). In the existence of contradicting evidence, here we report the observed presence of a statistically significant association between STH infections and geophagia among pregnant women in Jimma Town, Southwest Ethiopia. This findings corroborates with similar studies conducted in Kenya and Tanzania (36,39). The contradicting results might be due to the fact of the variation of STHs prevalence from region to region, and presence or absence of favorable soil environment for the existence of STHs eggs (34,40).

Moreover, our study has not determined the existence of STHs eggs from the soil samples claimed by the pregnant women for geophagic purpose. In the absence of soil analysis for the eggs of *A. lumbricoides* and *T. trichiura*, it may be difficult to determine the causal relationship and/or established association of STH infections and geophagia. However, studies conducted elsewhere documented the presence as well as the absence of significant association by analyzing both stool and soil samples (40). Therefore, we recommend detailed research on the effect of geophagia for STH infections in Ethiopia and integrated intervention to avoid pica practice, particularly during pregnancy and childhood.

The study has not determined the presence of infective stages of STHs in the soils and other non-food substances and anemia was not analyzed among pregnant women. Moreover, lack of precise information when our study participant did start eating soil before the study time limited our interpretation of the observed result related to the trimester stages.

In conclusion, the prevalence of STHs and geophagia among pregnant women was 19.7% and 17.4%, respectively. Soil-transmitted helminthic infections were significantly associated with geophagic pregnant women. Moreover, Pregnant women at their third trimester had more likely to eat soil intentionally. Further in-depth study including the analysis of the different consumed soil for STHs contamination is recommended. Health information for pregnant women on possible risks of STH infections from practicing geophagia should be given during ANC visits.
